# Seroprevalence and risk factors of Hepatitis E Virus infection among pregnant women in Addis Ababa, Ethiopia

**DOI:** 10.1371/journal.pone.0180078

**Published:** 2017-06-26

**Authors:** Meseret Abebe, Ibrahim Ali, Samuel Ayele, Johakim Overbo, Abraham Aseffa, Adane Mihret

**Affiliations:** 1 Armauer Hansen Research Institute, Addis Ababa, Ethiopia; 2 Addis Ababa University, College of Health Sciences, Addis Ababa, Ethiopia; 3 Norwegian Institute of Public Health, Oslo, Norway; Centers for Disease Control and Prevention, UNITED STATES

## Abstract

**Background:**

Hepatitis E virus (HEV) is highly endemic in several African countries with high mortality rate among pregnant women. The prevalence of antibodies to HEV in Ethiopian pregnant women is not known. The study was conducted to investigate the prevalence of anti-HEV IgG and anti-HEV IgM among pregnant women.

**Material and methods:**

A total of 386 serum samples were collected from pregnant women between April 2014 to January 2015 in Gandhi Memorial Hospital and four selected Health centers in Addis Ababa, Ethiopia. Data were collected for socio demographic characteristics using a structured questionnaire. Serum samples were examined for anti-HEV IgG and anti- HEV IgM using ELISA. The association of anti-HEV status with risk factors was assessed. Factors demonstrating significant association in bivariate analysis were included in multivariate logistic regression models. Analyses were performed using SPSS version 21.

**Results:**

Anti- HEV IgG antibody was detected in 122 (31.6%) women and two women (0.5%) were positive for anti-HEV IgM from the total 386 women. Age and educational status had statistically significant association with HEV infection. There was no significant association between anti-HEV antibody seroprevalence rate with trimester, parity, HIV status and other risk factors.

**Conclusion:**

In this study we found a high seroprevalence rate of anti-HEV IgG among pregnant women in Addis Ababa Ethiopia. Preventive measures like improvement of education and creating awareness may reduce the risk in pregnant women. Moreover nationwide surveillance of HEV especially in rural setting should be conducted to establish a national estimate and validate our findings.

## Introduction

Hepatitis E virus (HEV) is a small non-enveloped, positive-sense single-stranded RNA virus. It has been classified as the single member of the genus Hepevirus and has a similar structure to the viruses of the Caliciviridae and Tombusviridae families. This virus was first discovered during an outbreak in New Delhi, India, in 1955 [1–3]. Although hepatitis E virus (HEV) is sometimes referred to as an emerging infectious agent, it is well-established as a major cause of acute viral hepatitis (AVH) worldwide. An estimated one-third of the world’s population has been infected with HEV [4]. Of the more than 20 million infections estimated to occur globally each year, ~70,000 infections result in death. The vast majority of these deaths occur in resource-poor countries in Asia, Africa, and Latin America [5].

HEV infection can lead to more severe, acute liver disease in pregnant women and sometimes progress to fulminant hepatic failure (FHF) [6]. It also leads to severe complications which may result in fetal and/or maternal mortality, abortion, premature delivery, or death of a live-born baby soon after birth [7, 8].

HEV is transmitted primarily by the fecal–oral route [9].Vertical transmission of HEV from a pregnant woman to unborn fetus is very well documented [10]. The risk factors for HEV infection are related to resistance of HEV to environmental conditions, poor sanitation in large areas of the world, and HEV shedding in feces [11]. Following an incubation period of 2 to 6 weeks, an initial short lived IgM response is followed by longer-lasting IgG antibodies. The presence of anti-HEV IgM is a marker of acute infection and increased titers of anti-HEV IgG can indicate recent HEV infection [12].

In Ethiopia, according to a report in the early 1990s, which is the only available study on HEV, the HEV antibody seroprevalence observed in jaundice patients was 93%, pregnant women (59%) and healthy adults (3%). In this study, the occurrence of HEV during pregnancy was associated with high maternal and fetal morbidity and mortality [13]. Since there is no recent study on seropevalence of HEV among pregnant women in Addis Ababa, Ethiopia, it is important to have up to date data on seroprevalence of HEV among pregnant women.

## Material and methods

### 2.1 Study setting and period

The study was conducted in Addis Ababa in one public hospital (Ghandi Memorial Hospital) and four health centers (Bole 17, Woreda 23,Woreda 3 and Arada health centers.Gandhi memorial Hospital and those four Health centers were selected from 96 health centers and 6 public hospitals using a lottery method. Gandhi memorial Hospital is a maternal referral Hospital under the Addis Ababa city government health bureau and is located in the central part of Addis Ababa, Kirkos sub city. The hospital gives 10–15 delivery services per day. Bole 17, Woreda 23, Woreda 3 and Arada health centers are under Addis Ababa city government health bureau and they are found in Bole, Addis Ketema, Nifas silk lafto and Arada sub cities respectively in Addis Ababa. The health centers provide anti-natal care, delivery services, post-natal care services, ART service and other health services. In the health centers 8–12 new pregnant women attended ANC daily. The study was conducted between April 2014 and January 2015.

### 2.2 Study population and sample size

We used a convenient sampling technique and a total 386 pregnant women aged 16–40 years were included in the study. The study populations were all consecutive pregnant women who attended ANC clinic for check-up services at the study facilities. Pregnant women with no history of jaundice and who gave consent to participate in the study were evaluated by a questionnaire which dealt with information about socio demographic data (age, place of residence, marital status educational level, profession, source of water, sanitary condition and pet owner ship).

Sample size was assigned to each selected health facility proportional to the number of pregnant women who had follow up in the facility. Sixty pregnant women were enrolled from each health centers and 144 pregnant women were recruited from Gandhi memorial Hospital.

### 2.3 Ethical considerations

The proposal was approved by Armauer Hansen Research Institute (AHRI) Ethical Review Committee, Research and Ethical Review Committee of the Department of Medical Laboratory Science Collage of Health Sciences Addis Ababa University and Addis Ababa city government health bureau. Permission was also obtained from the Hospital and Health centers administrators. Written informed consent was sought from all study participants.

### 2.4 Data collection and processing

After their consent pregnant women who fulfilled the eligibility criteria were interviewed by trained data collectors using a pre-tested standard questionnaire to gather data on sociodemographic and risk factors.

### 2.5 Laboratory analysis

Five milliliter of blood was collected from each participant. Serum was separated at the study sites serum and transported to AHRI and kept at -20 0C until processing. HEV ELISA was performed at the Immunology Laboratory of Armauer Hansen Research Institute. All of the sera were screened in duplicate for IgG and IgM to HEV using ELISA Kit (WANTAI HEV Ab ELISA China) which shows high sensitivity and specificity compared to other assays including molecular methods [4].

Positive and negative controls were included in all the ELISA microplates. The presence or absence of HEV IgG and HEV IgM in the sample was determined by the amount of color intensity. The amount of color intensity is proportional to the amount of antibody captured in the wells, and to the amount of antibody in the sample respectively.

Specimens giving a value less than the Cut-off value were negative for this assay, which indicates that no anti-HEV IgG/HEV IgM antibodies have been detected with WANTAI HEV ELISA; therefore there are no serological indications for infection with HEV (A / C.O. < 1). Specimens giving a value equal to or greater than the Cut-off value were considered initially reactive, (A / C.O. ≥ 1) which indicates that anti-HEV IgG/HEV IgM antibodies have probably been detected with WANTAI HEV ELISA. Specimens with A value to Cut-off ratio between 0.9 and 1.1 were considered borderline and retested to confirm the initial test result.

### 2.6. Data analysis

The association of baseline anti-HEV status with potential risk factors was assessed. Factors demonstrating significant association in bivariate analysis were included in multivariate logistic regression models value < 0.05 was considered as statistical significance. Analyses were performed using SPSS version 21.

## Result

### 3.1 Socio demographic characteristics

A total of 386 pregnant women were screened for the presence of anti-HEV IgG and anti-HEV IgM antibodies. Their age ranged from 16 to 40 years, with a mean age ± SD of 28.9 ± 5.76 years. Among the study sparticipants, 90.4% were married and 59.8% of them were 26–35 years of age. Only 32(8%) pregnant women were illiterates. A Sociodemographic characteristics of the participants is presented in Table 1.

**Table 1:** Sociodemographic characteristics of study participants.

Socio-demographic Characteristics	Frequency
Age	
< = 25	141(36.5)
26–35	231(59.8)
> = 36	14 (3.6)
Religion	
Christian	315 (81.6)
Muslim	61 (15.8)
Don’t response	10 (2.6)
Marital status	
Married	349 (90.4)
Single	28 (7.3)
Divorced	5 (1.3)
Cohabiting	4 (1)
Educational status	
No formal education	32 (8.3)
Primary	113 (29.3)
Secondary	137 (35.5)
College and above	104 (26.9)
Occupation	
Government	55 (14.2)
Self employed	129 (33.4)
House wives	180 (46.6)
Student	22 (5.7)

### 3.2 Magnitude of HEV infection

The overall HEV sero-prevalence among pregnant women recruited in to the study over the 6 month period was 31.6%. Of the total study participants, 0.5% (2 out of 386) were anti-HEV IgM and anti-HEV IgG positivewhereas 31.6% (122 out of 386) were anti-HEV IgG positive.

### 3.3 Risk factors associated with HEV infection

**Fig 1:**
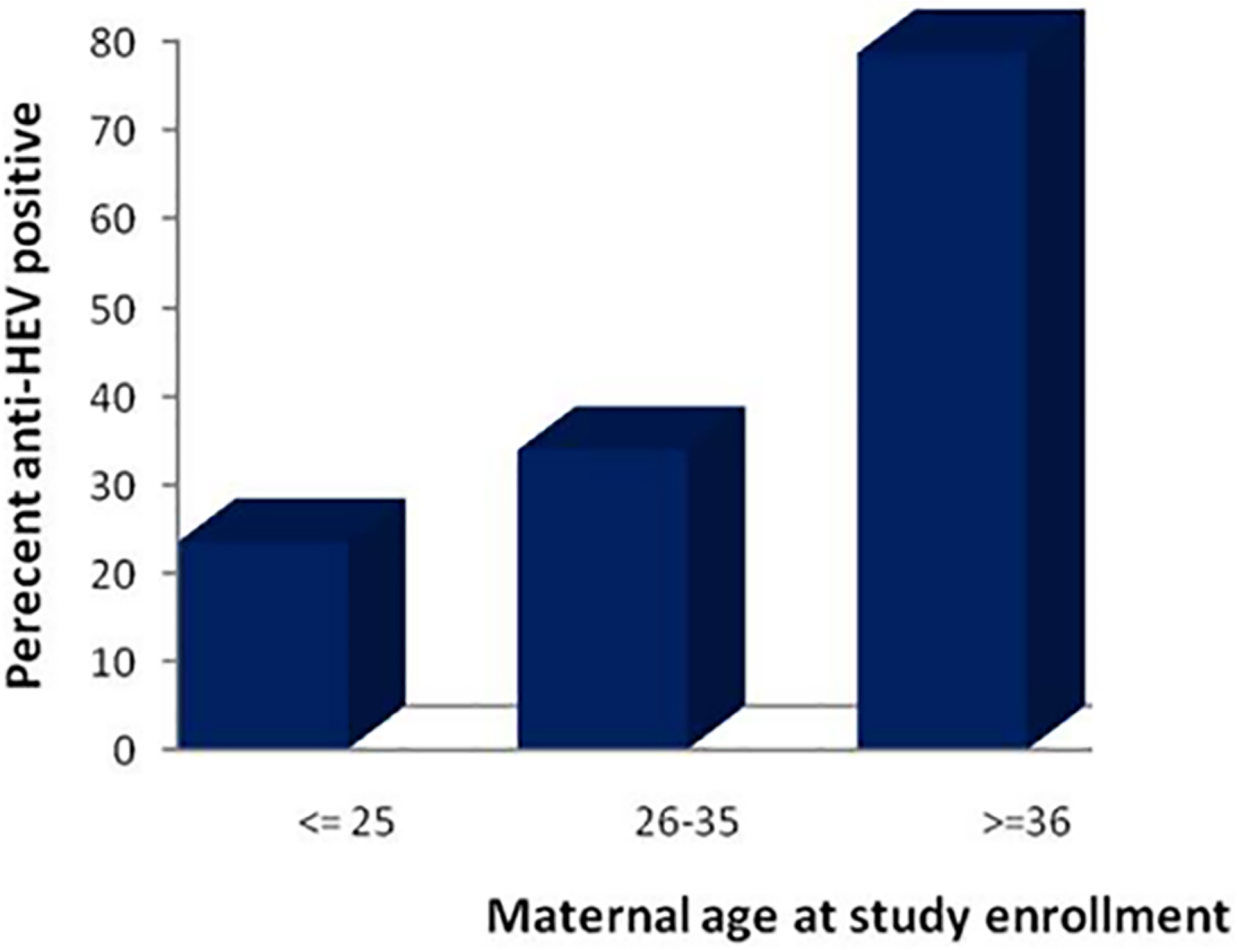
Anti-HEV seroprevalence stratified by age.

Anti-HEV seroprevalence had a strong association with age of the participant. The overall prevalence rate of antibodies to HEV was highest (`78.5%) among pregnant women ≥ 36 year group (p = 0.001), followed by (33.7%) in 26–30 years of age (p = 0.047), then (23%) in ≤ 25 year group (Fig 1).

There was a strong statistical association between educational level and HEV seroprevalence. Anti-HEV reactivity among pregnant women with primary education (36%; 41/113) (p = 0.010), was higher than that of their counterparts with secondary (35%, 49/137) (p = 0.049), and college and above (22%; 23/ 104) level of education.

In multivariate analysis, parity was not significantly associated with HEV infection, but showed an association in bivarate analysis (95% CI (1.385–9.143) p = 0.008). Highest rate of infection (55%) was seen in pregnant women who have two children and above compared with those who have one child and pregnant women with first pregnancy experience.

When potential risk factors were adjusted using multivariate analysis age and educational level remained the only significant factors. Our data did not show any statistical significant association between HEV infection with periods of pregnancy, blood transfusion, marital status, HIV status, washing hand after defecation and pet ownership (Table 2).

**Table 2:** Risk factors associated with maternal anti-HEV: bivariate and multivariate analysis.

Variable	Total	HEV IgG sero status		COR (95%CI)	p -value^a^	AOR(95% CI)	p- value^b^
		Pos n(%)	Neg n (%)				
**Age**							
< = 25	141	33(23.4)	108(76.6)	1			
26–35	231	78(33.8)	153(66.2)	1.68 (1.04–2.69)	0.035^*^	1.68(1.07–2.82)	0.047^*^
>36	14	11(78.6)	3(21.4)	12 (3.16–45.6)	0.000^*^	9.88 (2.43–40.12)	0.001^*^
**Trimester**							
First	129	40(31)	89(69)	1			
Second	137	49(35.8)	88(64.2)	1.18(0.69–2.05)	0.544		
Third	120	33(27.5)	87(72.5)	1.46(0.86–2.45)	0.157		
**Marital status**							
Married	349	113 (32.4)	236(67.6)	1			
Single	28	6(21.4)	22(78.6)	0.48(0.06–3.44)	0.464		
Cohabited	4	2(50)	2(50)	0.27(0.03–2.36)	0.238		
Divorced	5	1(20)	4(80)	0.25(0.01–4.73)	0.355		
**Education level**							
Illiterates	32	9(28.1)	23(71.9)	1.37(0.56–3.38)	0.484	1.23(0.53–3.36)	0.530
Primary	113	41(36.3)	72(63.7)	2.0 (1.09–3.65)	0.023^*^	2.25(1.21–4.19)	0.010^*^
Secondary	137	49(35.8)	88(64.2)	1.96(1.09–3.50)	0.023^*^	1.82(1.00–3.31)	0.049^*^
College and above	104	23(22.1)	81(77.9)	1			
**Parity**							
1^st^pregnancy	176	45(25.6)	131(74.4)	1			
1–2	185	64(34.6)	121(65.4)	1.54(0.97–2.42)	0.063	1.22(0.78–2.00)	0.421
2–3	20	11(55)	9(45)	3.55(1.38–9.14)	0.008^*^	2.23(0.81–6.17)	0.120
Morethan3	5	2(40)	3(60)	1.94(0.31–11.9)	0.475	0.79(1.08–5.82)	0.821
**HIV status**							
Positive	18	6(33.3)	12(66.7)	1.08(0.39–2.96)	0.872		
Negative	368	116(31.5)	252(68.5)	1			
**Blood Transfusion**							
Yes	17	7(41.2)	10(58.8)	1.55(0.57–4.16)	0.389		
No	369	115(31.2)	254(68.8)	1			
**Wash hand**							
Yes	372	115(30.9)	257(69.1)	1			
No	14	7(50)	7(50)	2.23(0.76–6.51)	0.141		
**Pet ownership**							
Yes	104	36(34.7)	68(65.3)	1.16(0.71–1.87)	0.548		
No	282	88(31.3)	194(68.7)	1			

*significant at p<0.05, COR-crude odds ratio, ADR-Adjusted odds ratio, p -value^a^ for COR, p–value^b^–for AOR, 1-logical reference

## Discussion

Hepatitis E virus is a major cause of liver disease worldwide, and although long-term sequelae are rare, the disease carries appreciable mortality in pregnant women [14].

Our study showed that the overall seroprevalence of HEV infection among study population in selected health facilities, Addis Ababa Ethiopia was (31.6%) which is comparable with a study done in Darfur, Western-Sudan where the seroprevalence of hepatitis E virus in pregnant women was 31.1% [15]. Our finding was higher than the result of similar studies done in BurkinaFaso (11.6%), Gabon (14.1%) and Ghana (28.6%) [16, 17, 18], but lower than the sero-prevalence of HEV infection among pregnant women in Egypt (84.3%), Sudan (41%) and India (33.6%) [19, 20, 21].

In the developed world the rate is significantly low. A study in Spain by Lindemann *et al* on 1040 pregnant women reported the rate of anti-HEV IgG was 3.6%. Prevalence of HEV IgG was found to be 7.7% and 10% in pregnant women in France and China, respectively which is much lower than our finding [22–24]. Regarding anti-HEV IgM antibodies, 0.5% (2 /386) of pregnant women were positive. Other have reported prevalence rate of 0% in France, 0.64% in Spain and 10% in Ghana [24, 23, 17].

Reason for these differences could be due to difference in level of hygiene, educational status, social status, endemicity of virus, different lifetime exposures of the participants to HEV and use of different test systems with varying sensitivity. For example in Egypt a study found higher HEV seroprvalence among pregnant women (84.3%). They suggested that reasons for high HEV sero prevalence could be the result of early childhood HEV exposures, producing long-lasting immunity and/or modify subsequent responses to exposure [20].

Mansuy et al. recently reported 53% prevalence of HEV antibodies in blood donors in southwestern France [25], a figure considerably higher than the 17% prevalence reported earlier for the same geographic region, when a different test system was used [26].

We found a significant association between age and higher anti-HEV positive values (p<0.05) which was consistent with a study done by Stoszek *et al*. [20]. Our findings showed that the odds of pregnant women whose age was between 26–34 being infected with HEV is 1.68 times higher than the odds of pregnant women whose age was < 25 years. The strong association between age and HEV seroprevalence in our study most likely reflects cumulative lifetime exposure to the virus.

Most studies demonstrated role of education in decline of HEV seroprevalence, and prevalence of hepatitis E in educated women is significantly low ([19,27] which was in agreement with our study, where women with college and above educational level have the lowest seropositivity from all study participants (22% 23/104) and The odds of pregnant women who have a primary educational status infected with HEV was 2 times higher than the odds of pregnant women who had a college and above educational status.

HEV infections are spread mainly by the faecal-oral route [28], but we found that washing hands with soap after defecation has no significant association with seropositivity. This could be due to the fact that the questionnaire used for the study may have been susceptible to responder bias. For example, as is common in many settings, proper hygiene and sanitation practices may have been over reported by respondents.

A study conducted in Northern India has shown that the low socio-economic status of pregnant women appeared to be the only risk factor associated with HEV seropositivity [19], but in our study it was difficult to assess the hypothesis that correlates income with seropositive status., because most of the pregnant women were not volunteer to fill their income on the questionnaires. But the high prevalence of HEV infection in pregnant women in our study might be due to the fact that in the study the samples were collected from governmental health institutions in which medical care is free or very cheap and therefore women of low socio-economic status frequently attend and our study population may lack a portion of pregnant women with higher socioeconomic and educational level.

Documented direct evidence for transfusion transmitted HEV infection has been reported [29]. But, in our study we found no significant association, this could be due to the fact, in our study only 4% (17/386) of the participants has received blood previously, so it is difficult to determine the association of the variable with HEV seropositivity with this insufficient data.

Regarding to HEV IgM antibody, since only two study participants were HEV IgM positive it was impossible to perform statistical analysis. Both of HEV IgM positive pregnant women had similar age which was 28 years. They were married and at their first trimester of pregnancy. One of HEV IgM positive study participant was HIV positive.

## Conclusion

In conclusion, the seroprevalence of HEV among pregnant women in this study is high. Preventive measures including improvement of education should be taken to decrease the spread and transmission of HEV. Since the finding of this study was limited by including only government health Institutions in urban setting and there was also lack of molecular techniques (HEV RNA detection) for confirmation of our results, more extensive studies should be conducted to evaluate the seroprevalence, to characterize the circulating HEV genotypes and to determine the current pathological and risk status in the general population of Ethiopia. However, the findings of this study have adequately shed light into the problem of Hepatitis E infection among pregnant women in Ethiopia.
